# EEG Classification for Hybrid Brain-Computer Interface Using a Tensor Based Multiclass Multimodal Analysis Scheme

**DOI:** 10.1155/2016/1732836

**Published:** 2016-01-03

**Authors:** Hongfei Ji, Jie Li, Rongrong Lu, Rong Gu, Lei Cao, Xiaoliang Gong

**Affiliations:** ^1^Department of Computer Science and Technology, Tongji University, No. 4800 Caoan Highway, Shanghai 200092, China; ^2^Department of Rehabilitation, Huashan Hospital, Fudan University, No. 12 Wulumuqi Middle Road, Shanghai 200040, China

## Abstract

Electroencephalogram- (EEG-) based brain-computer interface (BCI) systems usually utilize one type of changes in the dynamics of brain oscillations for control, such as event-related desynchronization/synchronization (ERD/ERS), steady state visual evoked potential (SSVEP), and P300 evoked potentials. There is a recent trend to detect more than one of these signals in one system to create a hybrid BCI. However, in this case, EEG data were always divided into groups and analyzed by the separate processing procedures. As a result, the interactive effects were ignored when different types of BCI tasks were executed simultaneously. In this work, we propose an improved tensor based multiclass multimodal scheme especially for hybrid BCI, in which EEG signals are denoted as multiway tensors, a nonredundant rank-one tensor decomposition model is proposed to obtain nonredundant tensor components, a weighted fisher criterion is designed to select multimodal discriminative patterns without ignoring the interactive effects, and support vector machine (SVM) is extended to multiclass classification. Experiment results suggest that the proposed scheme can not only identify the different changes in the dynamics of brain oscillations induced by different types of tasks but also capture the interactive effects of simultaneous tasks properly. Therefore, it has great potential use for hybrid BCI.

## 1. Introduction

Brain-computer interface (BCI) system, also known as a brain-machine interface, is designed to translate human brain signals into commands to control an external device [1]. It provides a direct communication pathway between human brain and computer or machine and is most helpful to people with severe motor disabilities [2]. Since the scalp-recorded electroencephalogram (EEG) has an exquisite temporal resolution in the order of millisecond and is a noninvasive brain signal measurement, it has been the most popular sensory signal used for BCI [3].

EEG-based BCI systems usually utilize changes in the dynamics of brain oscillations for control, such as event-related desynchronization/synchronization (ERD/ERS) with imagined movements, steady state visual evoked potential (SSVEP), P300 evoked potentials, and related components [4, 5]. Conventional BCIs typically use only one of these signals and have not been practically applicable because of the lack of reliability, low accuracy, and low information transfer rate (ITR) [6]. Therefore, there is a recent trend to combine different types of BCIs to create hybrid BCI, which could help improve ITR, usability, accuracy, speed, and other features. Pfurtscheller et al. took a motor imagery- (MI-) based BCI as a self-paced brain switch to initiate a four-step SSVEP-based BCI [7]. Li et al. designed hybrid BCI systems that combined P300 potential and MI-based BCIs for multidimensional control, for example, 2D cursor control [8] or the direction and speed control of a wheelchair [9]. Wang et al. introduced a hybrid BCI paradigm using P300 and SSVEP could significantly improve the classification accuracies and ITR [10]. Allison et al. confirmed that by detecting MI and SSVEP simultaneously classification reliability can be improved [5]. Our previous studies [11, 12] proposed a hybrid BCI combining SSVEP and MI-based ERD, and the system could generate parallel multidegree commands for real wheelchair navigation. More and more studies show hybrid BCIs, especially those working in a simultaneous manner [12–19], can reduce disadvantages of each conventional BCI system and increase efficiency and flexibility of control by detecting two or more different types of BCI tasks.

In EEG-based BCI, distinctive patterns induced by specific mental task can be identified and utilized for information transmission by the EEG classification algorithm. Effective and accurate feature extraction and classification are of paramount importance for the success of the BCI [2]. According to the characteristics of applied signals, conventional BCIs rely on time-frequency or temporal-spatial analysis for feature extraction and classification. For example, the most commonly used techniques in time-frequency analysis include Fourier transforms (FT) [20], autoregressive (AR) models [21], wavelet transform [22], and canonical correlation analysis (CCA) [23], while the temporal-spatial techniques typically used are independent component analysis (ICA) [24] and common spatial patterns (CSP) [25]. Although many signal analysis methods have been well developed in conventional BCI research, there is no algorithm focusing on the hybrid task classification. Now, in the hybrid BCI systems, different types of tasks are detected separately. In this case, EEG data collected in hybrid tasks are divided into individual groups and fed into separate processing procedures [7–19]. For example, in the hybrid BCIs combining SSVEP and MI, including our previously developed hybrid BCI [11, 12], the data from occipital channels were selected for SSVEP tasks, and the spectral features or other related features (e.g., CCA coefficients) were calculated to identify the SSVEP target. On the other way, specific algorithms to detect the ERD phenomena (e.g., CSP algorithm) were applied in the data from the parietal channels to discriminate the MI tasks [12, 14, 18, 19]. As a result of separate processing procedures, the interactive effects were ignored when different types of tasks were executed simultaneously. Therefore, although those methods could achieve reasonable results, they are not optimized for hybrid tasks classification.

There is an increased interest to represent EEG data as a multiway array named tensor, and tensor decomposition can be applied to exploit the characteristics of data among multiple modes [26–29]. Our previous work [30–32] proposed several tensor based schemes for EEG classification, in which single trial EEG data were denoted by multiway tensors and various tensor decomposition methods were proposed for multimodal analysis. However, those previous schemes were all designed for binary classification in conventional BCIs and cannot be applied in hybrid BCI as the number of the tasks in the hybrid paradigm increases greatly. Take our previously developed hybrid BCI [12], for example; the number of the hybrid tasks is the sum of the number of individual tasks and the number of simultaneous tasks, and that is fourteen for our hybrid BCI combining of two-class MI and four-class SSVEP. In particular, in this case, the tensor decomposition methods presented in the previous schemes would produce numerous tensor components, which result in the fact that the significant components reflecting the interactive effects of simultaneous tasks are overwhelmed with lots of redundant ones.

Therefore, in this work, we propose an improved tensor based multiclass multimodal analysis scheme especially for hybrid BCI, in which EEG signals of the hybrid tasks are denoted as multiway tensors, a nonredundant rank one tensor decomposition model is proposed to obtain nonredundant tensor components, a weighted Fisher criterion is designed to select multimodal discriminative patterns among hybrid tasks without ignoring the interactive effects of simultaneous tasks, and support vector machine (SVM) is extended to multiclass classification for hybrid tasks. Applications in three datasets suggest that the proposed scheme can not only identify the different changes in the dynamics of brain oscillations induced by different types of tasks, but also capture the interactive effects of simultaneous tasks properly.

## 2. Tensor Based Multiclass Multimodal Analysis Scheme

**Figure 1:**
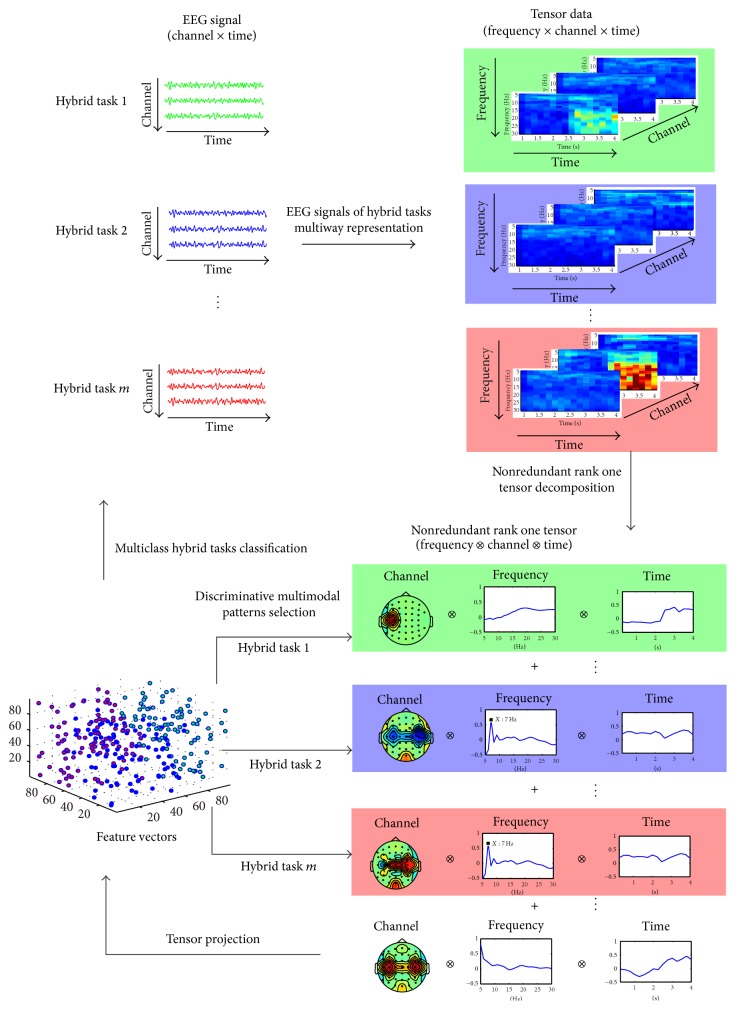
Tensor based multiclass multimodal analysis for hybrid tasks.

The proposed tensor based multiclass multimodal analysis scheme for hybrid BCI is illustrated in Figure 1. In this scheme, first, multichannel EEG data of hybrid tasks are transformed into multiway tensors representation in multimodes of channel, time, and frequency domain, and for each hybrid task, including individual tasks and simultaneous tasks, the assembled tensors in training dataset are calculated. Second, a nonredundant rank one tensor decomposition model is proposed to obtain the nonredundant rank one tensor components from the assembled tensors. Third, projection coefficients to the selected rank one tensors for each hybrid task are calculated and concatenated as the feature vectors. Fourth, a weighted Fisher criterion especially regarding the interactive effects of simultaneous tasks is proposed to select discriminative features and then obtain discriminative multimodal patterns among hybrid tasks. Finally, multiclass SVM is applied to multiclass classification for hybrid tasks. Each component is described in the following subsections.

### 2.1. EEG Signal of Hybrid Tasks Multiway Representation

Multichannel EEG signals can be added to the spectral modality by wavelet transform and yield a three-way tensor data (this step is conducted in the same way as proposed in our previous work [30, 31], and detailed description can be found in [30]). Let *X*_(*c*,*f*,*t*)_ denote the convolution amplitude with a wavelet, at *c*th channel, *f*th frequency, and *t*th time. Then for each class of hybrid tasks, the assembled tensors are calculated as follows:

(1) $$\begin{array}{c} {\bar{X_{\left(c,f,t\right)}}}^{i=1,\ldots ,m}=\overset{i}{\underset{j=1,\ldots ,n_{i}}{\sum }}X_{{\left(c,f,t\right)}_{j}}. \end{array}$$

Here, *i* = 1,…, *m* denotes the different class of hybrid tasks, and there are *n*_*i*_ trials for each class of hybrid tasks. Owing to the multiway representation, it becomes possible to exploit the different characteristics for different types of hybrid tasks simultaneously.

### 2.2. Nonredundant Rank One Tensor Decomposition

Tensor decomposition can analyze multiway data without losing some potential information among modalities [26]. Parallel factor analysis (PARAFAC) decomposition is a classic tensor decomposition model and has been successfully used as an exploratory tool in the analysis of EEG signals [28]. Compared to other popular tensor models, for example, Tucker model [33], nonnegative multiway factorization (NMWF) [29], the number of components included in the model can be directly limited and the patterns of components are easily interpretable [34], which are very important for EEG analysis for hybrid BCI with a large number of tasks. However, the components in the model are out of order. Besides, the given number of components greatly impacts on the decomposition results. Our previous work [32] proposed an ordered PARAFAC model to find a set of ordered tensor components. Although it ensures achieving a more stable model under mild conditions than classic PARAFAC model, redundant components increase dramatically with increasing number of tasks in the hybrid paradigm, which directly impacts further feature extraction for the hybrid tasks. Therefore, in order to overcome this issue, a nonredundant rank one tensor decomposition model is especially developed in this work.

For each class of hybrid tasks, the assembled tensor ${\bar{X_{\left(c,f,t\right)}}}^{i=1,\ldots ,m}$ is decomposed into sums of nonredundant rank one tensors by the proposed nonredundant rank one tensor decomposition model, which is described as shown in Algorithm 1.

### 2.3. Tensor Projection

By the proposed nonredundant rank one tensor decomposition, the assembled tensors ${\bar{X_{\left(c,f,t\right)}}}^{i=1,\ldots ,m}$ could be decomposed into sums of nonredundant rank one tensors. Each rank one tensor can be denoted as the outer product of the unit vectors. Let *u*_*j*_^1,*i*^○*u*_*j*_^2,*i*^○*u*_*j*_^3,*i*^ define the *j*th rank one tensor for the *i*th class of the hybrid tasks; then for each tensor data *X*_(*c*,*f*,*t*)_, projection coefficients to the nonredundant rank one tensors are calculated, respectively, as follows:

(2) $$\begin{array}{c} {c_{j=1,\ldots ,r}}^{i=1,\ldots ,m}=\frac{{\left(u_{j}^{1,i}○u_{j}^{2,i}○u_{j}^{3,i}\right)}^{T}X}{\left({\left(u_{j}^{1,i}○u_{j}^{2,i}○u_{j}^{3,i}\right)}^{T}\left(u_{j}^{1,i}○u_{j}^{2,i}○u_{j}^{3,i}\right)\right)}. \end{array}$$

Then those projection coefficients for each hybrid task are concatenated as the feature vectors.

### 2.4. Discriminative Multimodal Patterns Selection

As mentioned before, there is no algorithm focusing on the hybrid task classification. So far, EEG data of hybrid tasks are divided into individual groups and fed into separate processing procedures. In this case, the interactive effects on distinctive patterns are totally ignored when different types of tasks are executed simultaneously. Fisher score has been used to select discriminative features for binary classification in our previously proposed tensor based scheme [30]. Here, we propose a weighted Fisher criterion for multimodal patterns selection, in which greater weights are given to simultaneous tasks to help extract the distinctive patterns when different types of tasks are executed simultaneously rather than individually.

Let *c*_*j*_^*i*^ denote the *j*th  (1 ≤ *j* ≤ *N*_*i*_) feature in the *i*th  (1 ≤ *i* ≤ *C*) class, *m*_*i*_ = (1/*n*_*i*_)∑_*j*=1_^*n*^ *c*_*j*_^*i*^ is the mean value for this feature in the *i*th class, and *m* = (1/*n*_*i*_)∑_*i*=1_^*c*^ *n*_*i*_ *m*_*i*_ is the mean value for this feature in all classes. Given *c*_*j*_^*i*^, the corresponding weighted Fisher criterion is defined by the ratio of the weighted between-class variance to the within-class variance, as follows:

(3) $$\begin{array}{c} J_{j}=\frac{\sum_{i=1}^{c}\left(n_{i}/n\right)\times d_{i}{\left(m_{i}^{j}-m^{j}\right)}^{2}}{\left(1/n\right)\sum_{i=1}^{c}\sum_{}^{}{\left(x_{i}^{j}-m^{j}\right)}^{2}}, \end{array}$$

where *d*_*i*_ = 1 for each individual task and *d*_*i*_ = 1 + *τ* for each simultaneous task (*τ* is a positive parameter and is tuned during the training procedure for each subject). For the *i*th class of hybrid task, the weighted Fisher criterion ratios of features *c*_*j*=1,…,*N*_*i*__^*i*^ are computed, and then features with ratio greater than a given threshold value are retained as the discriminative features. The corresponding multiway subspaces of rank one tensors are selected as the discriminative multimodal patterns for this hybrid task. Given the greater weights, the interactive effects of simultaneous tasks would not be overwhelmed in the individual patterns when the number of tasks increases greatly in hybrid BCI.

### 2.5. Multiclass Hybrid Task Classification

SVM [35] has been applied to pattern classification in various fields and achieves good results due to its excellent generalization ability [36]. In BCIs, SVM also acquires top level performance [37–39]. For a linearly separable binary classification problem, SVM constructs a maximum-margin hyperplane to separate the two classes of samples. Moreover, SVM can also map samples into high-dimensional spaces and perform a nonlinear classification by kernel trick efficiently.

In this scheme, a multiclass SVM method is applied for hybrid task classification. For *k*-classes of hybrid tasks, we convert the multiclass classification into the two-class classifications by constructing *k*(*k* − 1)/2 binary classifiers. Each binary classifier solves the two-class classification problem between the hybrid tasks. By this approach, the multiclass classification for hybrid tasks is converted into the two-class classification. We choose the Gaussian radial basis function (RBF) as the kernel function, and the SVM parameters, *c* and *γ*, are selected by a grid search using 5-fold cross-validation in the training data.

## 3. Data Description

Three different types of EEG datasets collected in our hybrid BCI study experiment [12] were applied, and we added two more healthy male subjects afterwards using the same experimental setup. There were totally nine subjects in this work. During the data collection, they were required to perform individual MI tasks, individual SSVEP tasks, and hybrid tasks of MI and SSVEP, respectively. MI tasks were to imagine movements of the right hand or the left hand. The stimuli frequencies for SSVEP were 7 Hz, 8 Hz, 9 Hz, and 11 Hz. EEG signals were recorded by Gtec Amplifier with the sampling rate of 256 Hz and filter band of 5–30 Hz. The right earlobe was used as the reference. Fifteen electrodes, placed at FC3, FC4, Cz, C5, C3, C1, C2, C4, C6, CP3, CP4, POz, Oz, O1, and O2, following the 10–20 international system, were chosen in this experiment. For each subject, dataset 1 includes 120 trials (60 trials for each MI task; each epoch contains 2 seconds' data in noncontrol state and 2 seconds' MI task data), dataset 2 contains 30 seconds' continuous data for each stimulus, and there are 168 trials in dataset 3 (12 trials for each hybrid task; each epoch includes 2 seconds' data in noncontrol state and 2 seconds' hybrid task data). For more detailed description of the experiment and data, please see [12].

## 4. Data Analysis and Results Evaluation

In this section, first, the proposed scheme is applied in dataset 1 and dataset 2 to confirm its efficiency in conventional BCI tasks, and then it is applied to dataset 3 to evaluate its performance in hybrid BCI tasks.

### 4.1. Individual MI and SSVEP Task Classification

**Figure 2:**
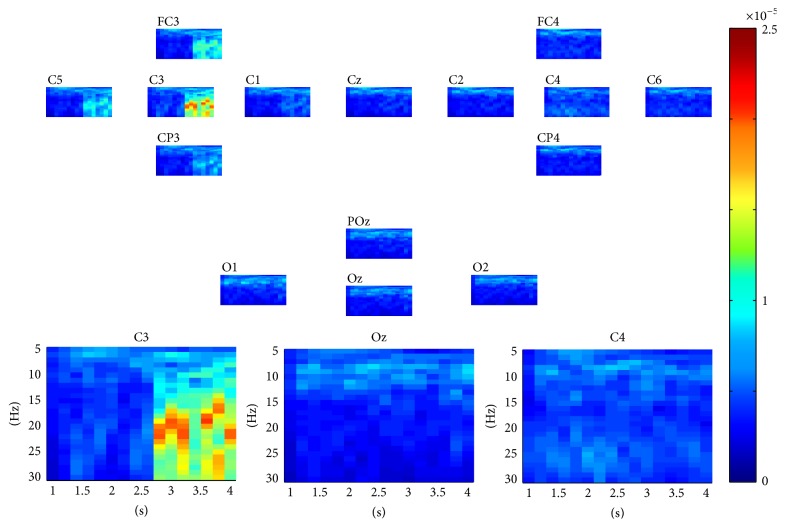
A visualization example of the assembled tensor for the left hand MI task. The spectrograms are shown at each channel according to channels distribution over scalp, with time ranging from 0 to 4 s and frequency ranging from 5 to 30 Hz. The spectrograms at C3, Oz, and C4 channels are enlarged in the bottom.

**Figure 3:**
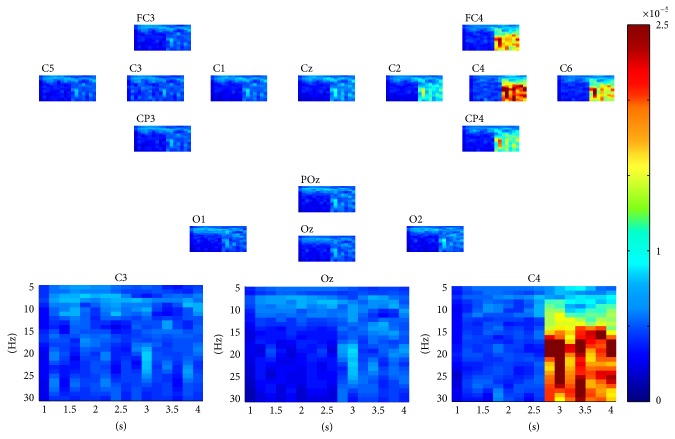
A visualization example of the assembled tensor for the right hand MI task. The spectrograms are shown at each channel according to channels distribution over scalp, with time ranging from 0 to 4 s and frequency ranging from 5 to 30 Hz. The spectrograms at C3, Oz, and C4 channels are enlarged in the bottom.

For each epoch in dataset 1, as described above, a three-way tensor was generated in multimodes of channel, time, and frequency. The frequency range was set to 5 Hz–30 Hz with 1 Hz spectral resolution and the time range was set to 1–4 s with 0.25 s temporal resolution. The assembled tensor for each MI task was calculated as described in Section 2. Figures 2 and 3 show visualization examples of the assembled tensors for right and left hand MI tasks by illustrating spectrograms at each channel, respectively. The power enhancements within *α* and *β* rhythms on the left/right motor cortex, especially at C3/C4 channels, can be seen when the subject began to execute the left/right hand MI tasks. The characteristics of the assembled tensors for MI tasks are exactly consistent with the ERD/ERS with imagined movements.

**Figure 4:**
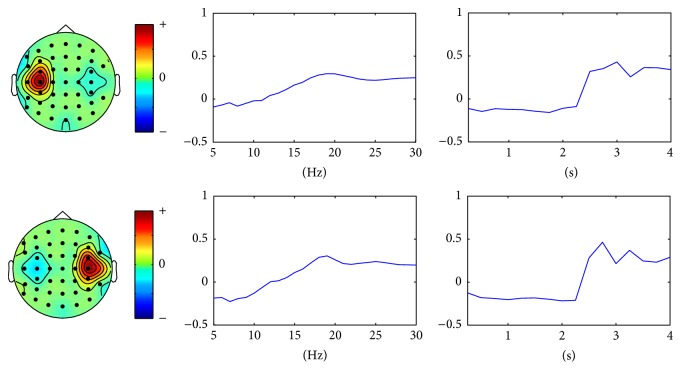
The multimodes of channel, time, and frequency of the two selected nonredundant rank one tensor components for MI tasks.

Figure 4 shows the multimodes of channel, time, and frequency of the two selected nonredundant rank one tensor components, namely, the discriminative multimodal patterns. The spatial, spectral patterns of two components match well with the difference of ERD/ERS for different hand MI tasks in the time-frequency domain. Furthermore, the temporal patterns show the amplitude of the two components increases rapidly after 2 s, which exactly matches with the fact that the MI task began at 2 s. It can be concluded that the proposed scheme reveals the changes in the dynamics of brain oscillations for MI tasks.

**Figure 5:**
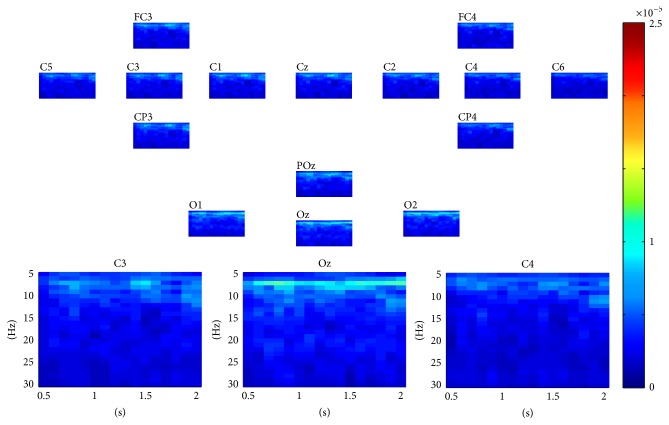
A visualization example of the assembled tensor for a SSVEP task (focusing on the 7 Hz stimulus). The spectrograms are shown at each channel according to channels distribution over scalp, with time ranging from 0 to 2 s and frequency ranging from 5 to 30 Hz. The spectrograms at C3, Oz, and C4 channels are enlarged in the bottom.

**Figure 6:**
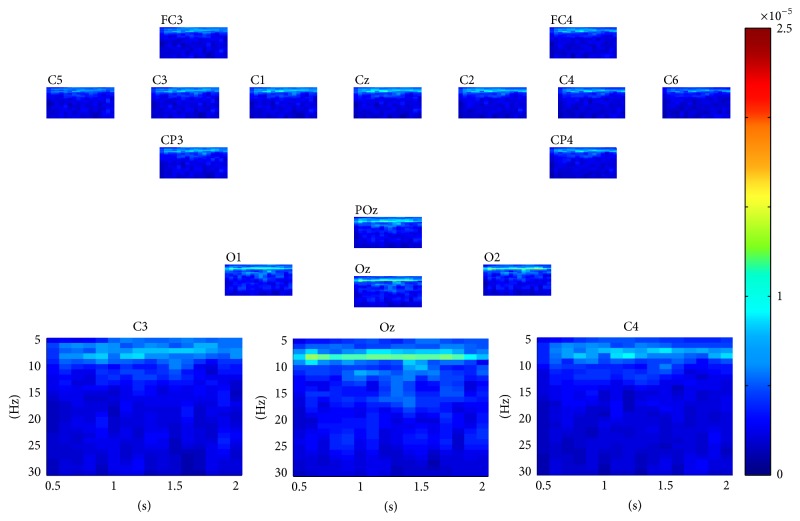
A visualization example of the assembled tensor for a SSVEP task (focusing on the 8 Hz stimulus). The spectrograms are shown at each channel according to channels distribution over scalp, with time ranging from 0 to 2 s and frequency ranging from 5 to 30 Hz. The spectrograms at C3, Oz, and C4 channels are enlarged in the bottom.

**Figure 7:**
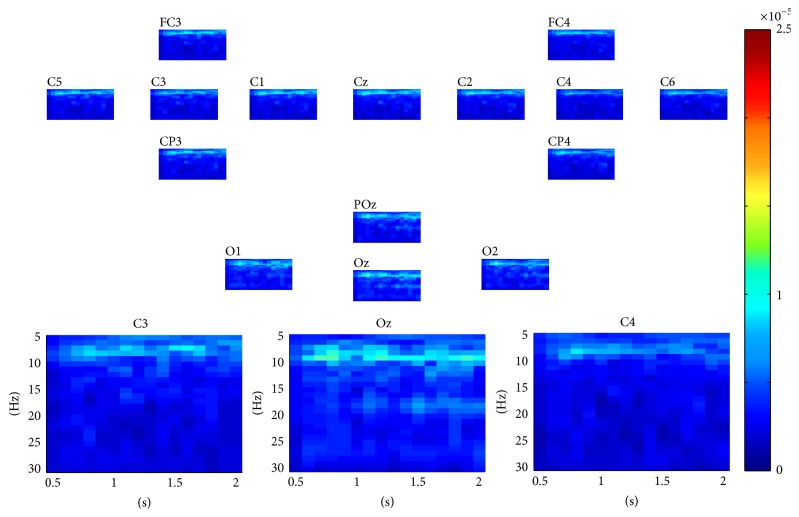
A visualization example of the assembled tensor of a SSVEP task (focusing on the 9 Hz stimulus). The spectrograms are shown at each channel according to channels distribution over scalp, with time ranging from 0 to 2 s and frequency ranging from 5 to 30 Hz. The spectrograms at C3, Oz, and C4 channels are enlarged in the bottom.

**Figure 8:**
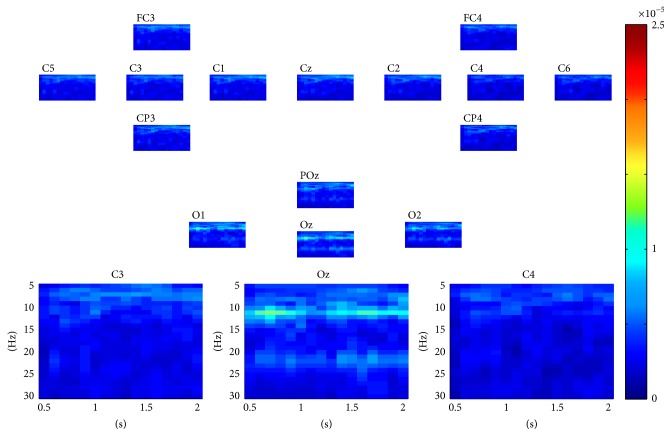
A visualization example of the assembled tensor of a SSVEP task (focusing on the 11 Hz stimulus). The spectrograms are shown at each channel according to channels distribution over scalp, with time ranging from 0 to 2 s and frequency ranging from 5 to 30 Hz. The spectrograms at C3, Oz, and C4 channels are enlarged in the bottom.

For SSVEP task classification, the continuous data in dataset 2 were segmented into 2 seconds' epochs. For each epoch, similarly, a three-way tensor was generated in multimodes of channel, time, and frequency. The frequency range was set to 5 Hz–30 Hz with 1 Hz spectral resolution and the time range was set to 1-2 s with 0.25 s temporal resolution. Then the assembled tensor for each SSVEP task was calculated. Figures 5, 6, 7, and 8 show visualization examples of the assembled tensors for each SSVEP task (focusing on the stimulus flickering at 7 Hz, 8 Hz, 9 Hz, and 11 Hz, resp.) by illustrating spectrograms at each channel. As can be seen in those figures, there are clear power enhancements at frequencies of 7 Hz, 8 Hz, 9 Hz, and 11 Hz and some corresponding second harmonics and at the channels on the occipital cortex, especially at Oz channel. The characteristics of the assembled tensors for SSVEP tasks are exactly consistent with the characteristics of corresponding SSVEP.

**Figure 9:**
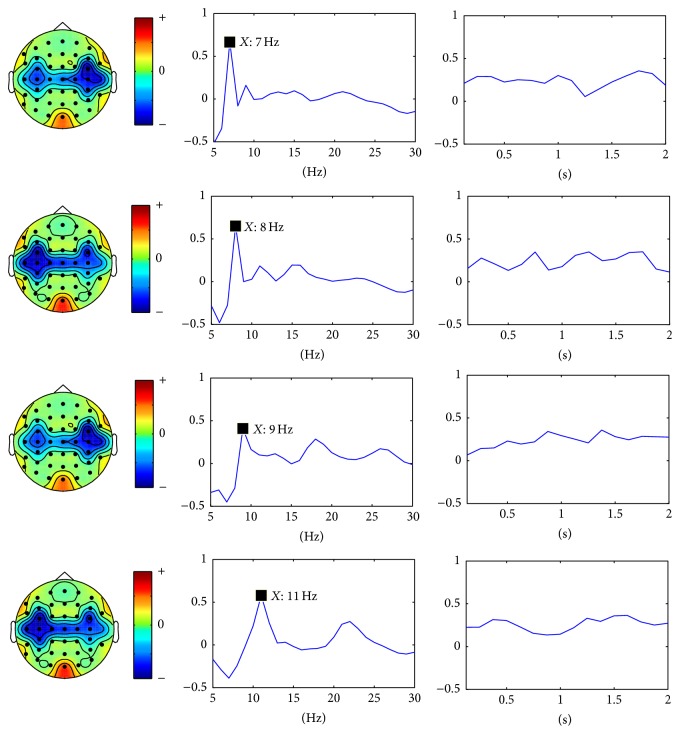
The multimodes of channel, time, and frequency of the four selected nonredundant rank one tensor components for SSVEP tasks.

Figure 9 shows the multimodes of channel, time, and frequency of the four selected rank one tensors by the proposed scheme. The corresponding spectral patterns match well the characteristics of corresponding SSVEP in the frequency domain. They show four evident peaks at frequencies of 7 Hz, 8 Hz, 9 Hz, and 11 Hz, respectively. Furthermore, the spatial patterns show the channels on the occipital cortex, especially Oz channel, possess the highest weight for SSVEP tasks. It can also be concluded that the proposed scheme reveals the changes in the dynamics of brain oscillations for SSVEP tasks.

The classification results in those two different types of datasets were compared with two other algorithms in BCIs, that is, CSP and CCA. CSP and CCA are highly successful in classifying MI and SSVEP tasks, respectively, and they are also the most commonly used methods in hybrid MI and SSVEP BCIs [12, 14, 18, 19]. For the CSP method, channels FC3, FC4, C5, Cz, C3, C1, C2, C4, C6, CP3, and CP4 on the motor cortex were chosen, while the channels POz, O1, O2, and Oz, on the occipital cortex were selected for the CCA method.

Table 1 summarizes the classification results of CSP, CCA and the proposed scheme for MI tasks and SSVEP tasks in conventional BCI. For each type of BCI task, the classification accuracies achieved by the proposed scheme are very close to those of CSP and CCA. For different types of tasks, with multimodal analysis, the proposed scheme performs as efficiently as CSP and SSVEP. Paired-sample *t*-tests show there is no significant difference between the proposed scheme and CSP (*P* = 0.8926) and proposed scheme and CCA (*P* = 0.8158). The results suggest the proposed scheme can identify different characteristic changes in the dynamics of brain oscillations, such as ERD/ERS and SSVEP, induced by different types of tasks in conventional BCIs.

### 4.2. Hybrid MI and SSVEP Task Classification

Above results confirm that the multiway tensor representation in multidomain of time, frequency, and channel can identify different characteristic changes for different types of tasks, such as MI-based ERD and SSVEP. Moreover, the proposed scheme is efficient in extracting multimodal discriminative patterns for different types of conventional BCI tasks. To investigate its performance in hybrid task classification, we further apply the proposed scheme to dataset 3.

Dataset 3 contains EEG data collected in hybrid MI and SSVEP tasks. For each epoch, a three-way tensor was generated in the previous manner. The frequency range was set to 5 Hz–30 Hz with 1 Hz spectral resolution and the time range was set to 1–4 s with 0.25 s temporal resolution. The assembled tensor for each class of hybrid task was calculated according to the method described in Section 2.

**Figure 10:**
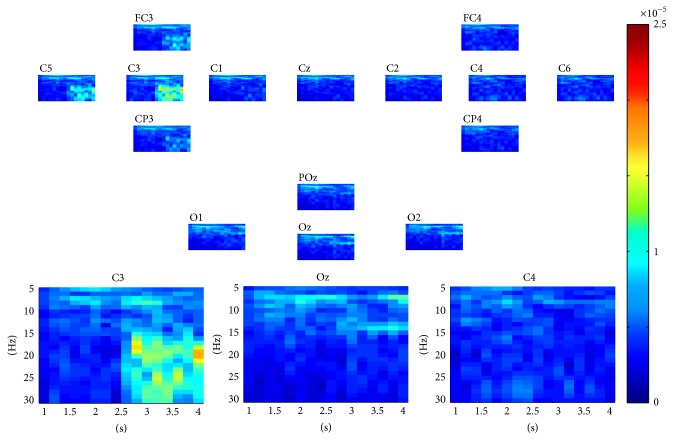
A visualization example of the assembled tensor for a hybrid of MI and SSVEP task (imagining left hand movements and focusing on the 7 Hz stimulus simultaneously). The spectrograms are shown at each channel according to channels distribution over scalp, with time ranging from 0 to 4 s and frequency ranging from 5 to 30 Hz. The spectrograms at C3, Oz, and C4 channels are enlarged in the bottom.

**Figure 11:**
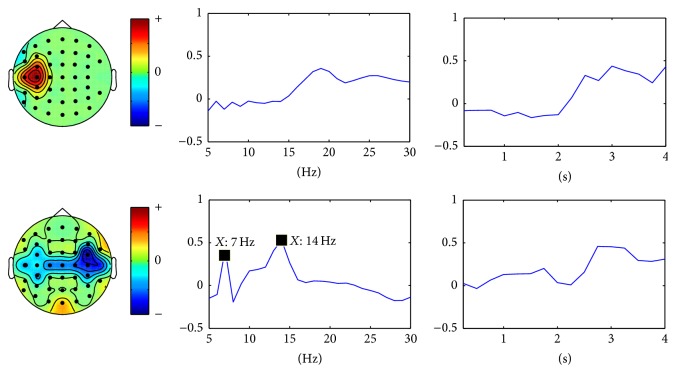
The multimodes of channel, time, and frequency of the two selected nonredundant rank one tensor components for the hybrid of MI and SSVEP task (imagining left hand and focusing on the 7 Hz stimulus simultaneously).

Figure 10 shows a visualization example of the assembled tensor for a hybrid task (imagining left hand movements and focusing on the 7 Hz stimulus simultaneously).  Figure 11 presents the multimodes of channel, time, and frequency of the two selected nonredundant rank one tensor components. As illustrated in Figure 10, the power on the left brain hemisphere increases from 2 s when the left hand MI task began to be executed, especially from 15 Hz to 30 Hz, at channel C3, and at the same time, the high power appears at channels on the occipital cortex, especially Oz, at 7 Hz and 14 Hz. Here, although the SSVEP should have the same frequency at fundamental frequency (7 Hz) and second harmonics frequency (14 Hz), considering that the assembled tensor presents the high power at 7 Hz before 2 s (in the noncontrol state), the power change at 14 Hz should be more significant for the SSVEP identification than 7 Hz in the hybrid tasks. The extracted discriminative patterns in Figure 11 match those spectral-temporal characteristics very well. Furthermore, because the left hand movement imagery was executing at the same time, which would induce the ERD on the contralateral hemisphere, that is, the power decrease within *α* and *β* rhythms on the right brain hemisphere, the power at 7 Hz and 14 Hz (within *α* and *β* rhythms) should decrease more greatly at channel C4 (on the right brain hemisphere) than C3 (on the left brain hemisphere). Compared to the discriminative patterns for individual 7 Hz SSVEP task (shown in Figure 9, the multimodes of the first rank one tensor), this spatial pattern (shown in Figure 11, the multimodes of the second rank one tensor) shows great imbalanced weights at the channels C3 and C4, which clearly reflects the interactive effects on discriminative patterns when those two different types of tasks were performed simultaneously in hybrid BCI.

**Figure 12:**
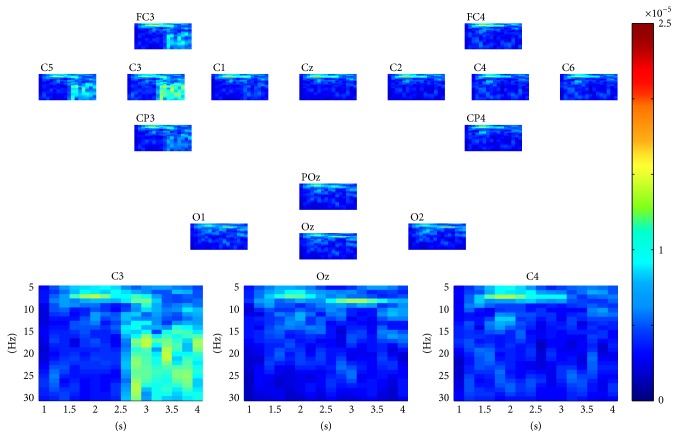
A visualization example of the assembled tensor for a hybrid of MI and SSVEP task (imagining left hand movements and focusing on the 8 Hz stimulus simultaneously). The spectrograms are shown at each channel according to channels distribution over scalp, with time ranging from 0 to 4 s and frequency ranging from 5 to 30 Hz. The spectrograms at C3, Oz, and C4 channels are enlarged in the bottom.

**Figure 13:**
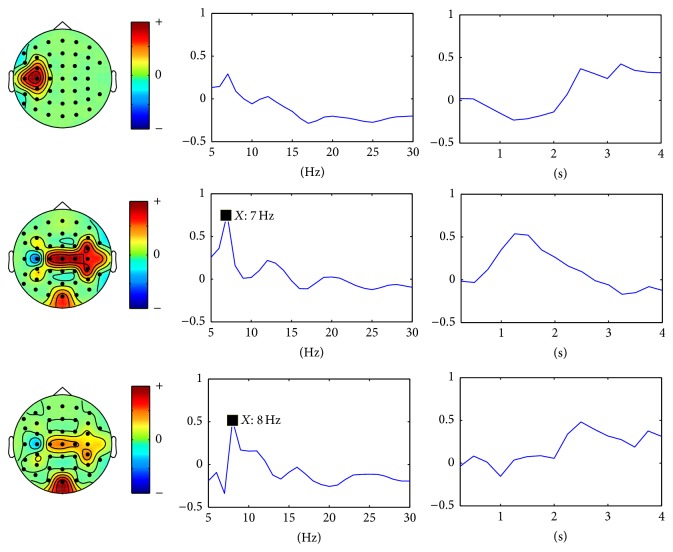
The multimodes of channel, time, and frequency of the three selected nonredundant rank one tensor components for the hybrid of MI and SSVEP task (imagining left hand and focusing on the 8 Hz stimulus simultaneously).

Figure 12 corresponds to a visualization example of the assembled tensor for another hybrid task (imagining left hand movements and focusing on the 8 Hz stimulus simultaneously). As previously reported, much high power appears at 7 Hz before 2 s (in the noncontrol state). However, the frequency of the high power is shown to change from 7 Hz to 8 Hz, especially at channels on the occipital cortex when the 8 Hz SSVEP task began. Meanwhile, the power at 7 Hz (within *α* rhythm) should also decrease at the channels on the right brain hemisphere due to the ERD produced by left hand movement imagery. The multimodes of the three selected rank one tensors are illustrated in Figure 13, and it is easy to find that the first and third rank one tensors correspond to the MI and SSVEP task identification, respectively. Particularly, the second rank one tensor reveals the power change at 7 Hz both on the right motor cortex and occipital cortex induced by the simultaneous SSVEP and MI tasks.

Those results show that there are some interactive effects when the different types of tasks are executed simultaneously and the proposed scheme could extract those patterns properly.

Table 2 summarizes the classification results for the hybrid tasks with the proposed scheme, CSP, and CCA. It should be pointed out that the proposed scheme was applied for hybrid task classification directly, which means it classified the MI and SSVEP in hybrid tasks simultaneously, while CSP can only be used for MI and CCA only for SSVEP. As can be seen, for more than half of subjects, the proposed scheme can obtain higher accuracies than the separate analysis method, that is, CSP and the CCA, and its average accuracies are better. It should be noticed that CSP and CCA performances drop dramatically for some of the subjects (e.g., CSP for sub. 8 and sub. 9; CCA for sub. 1, sub. 8, and sub. 9), while the proposed scheme can achieve much better results. We conducted *F*-tests to compare the performance differences in conventional task and hybrid tasks between the proposed scheme and the CSP method and the proposed scheme and the CCA method. *P* value = 0.0136 and *P* value = 0.0146 were obtained, respectively, which suggest their performance differences in conventional task and hybrid tasks are statistically significant. It can be concluded that the interactive effects of simultaneous tasks identified by the proposed scheme are helpful to the EEG classification in hybrid tasks.

## 5. Conclusion

In this paper, we propose an improved tensor based multiclass multimodal scheme especially for EEG analysis in hybrid BCI. Compared to current signal analysis methods for hybrid BCI, EEG data need not to be divided into individual groups and fed into the separate processing procedures. In this scheme, owing to tensor representation on multimodes of channel, time, and frequency, different characteristics of EEG signals can be presented simultaneously. Moreover, a nonredundant rank one tensor decomposition algorithm is proposed to obtain nonredundant rank one tensor components, and a weighted Fisher criterion is designed to select multimodal discriminative patterns among hybrid tasks without ignoring the interactive effects of the simultaneous tasks. Finally, SVM is extended to multiclass classification for hybrid tasks. Applications in three datasets suggest that the proposed scheme can not only identify the different changes in the dynamics of brain oscillations induced by different types of tasks, but also capture the interactive effects of simultaneous tasks.

## 6. Discussion

This work presents a novel method of EEG analysis for hybrid BCI by considering the interactive effect of the simultaneous tasks and demonstrates that it is helpful to improve the classification results for hybrid tasks. Although the proposed scheme is not suitable for online BCI because tensor generation and decomposition are very time-consuming, it is still very useful for developing hybrid BCI. It could help to learn the difference when two or more tasks are executed simultaneously rather than individually and obtain the multimodal information of the difference, including channel, time, and frequency. Taking advantage of the revealed multimodal information, some simple methods for online BCI could be improved and acquire better results. Therefore, the proposed scheme is a potential efficient tool in EEG analysis for hybrid BCI.

## Figures and Tables

**Algorithm 1:**
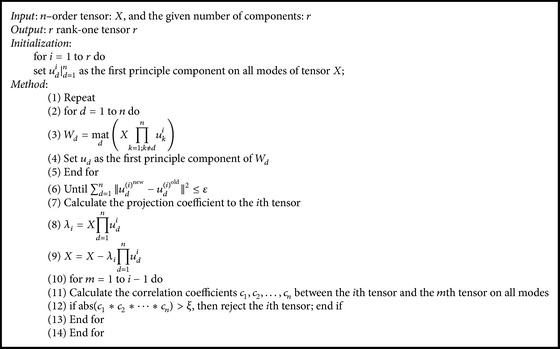
The nonredundant rank one tensor decomposition algorithm.

**Table 1:** Classification results (%) for conventional BCI tasks.

	Sub. 1	Sub. 2	Sub. 3	Sub. 4	Sub. 5	Sub. 6	Sub. 7	Sub. 8	Sub. 9	Avg.
CSP for MI	**79.2**	69.2	**100.0 **	**100.0 **	**50.8**	41.7	91.7	**88.7**	**67.5**	**76.5**
TbMMS for MI	72.1	**73.0**	99.1	98.7	50.0	**51.0**	**95.0**	83.6	64.2	76.3
CCA for SSVEP	**94.2**	57.5	**98.3**	93.8	87.6	**96.7**	**95.0**	**90.4**	**78.8**	**88.0**
TbMMS for SSVEP	92.1	**62.1**	97.4	**94.7**	**89.0**	94.0	94.3	89.8	77.3	87.9

TbMMS denotes the proposed tensor based multiclass multimodal analysis scheme.

**Table 2:** Classification results (%) for the hybrid BCI tasks.

	Sub. 1	Sub. 2	Sub. 3	Sub. 4	Sub. 5	Sub. 6	Sub. 7	Sub. 8	Sub. 9	Avg.
CSP for MI	**77.1**	75.0	**100.0 **	98.3	58.3	**53.3**	96.7	69.1	53.2	75.7
TbMMS for MI	73.1	**80.4**	**100.0**	**99.7**	**60.0**	51.0	**98.0**	**85.3**	**73.3**	**80.1**
CCA for SSVEP	82.0	**60.1**	97.5	90.3	90.0	**94.2**	98.0	81.3	63.9	84.1
TbMMS for SSVEP	**90.1**	58.0	**98.1**	**94.7**	**91.4**	94.0	**99.0**	**89.3**	**75.2**	**87.8**

TbMMS denotes the proposed tensor based multiclass multimodal analysis scheme.
